# QTL Characterization of Fusarium Head Blight Resistance in CIMMYT Bread Wheat Line Soru#1

**DOI:** 10.1371/journal.pone.0158052

**Published:** 2016-06-28

**Authors:** Xinyao He, Morten Lillemo, Jianrong Shi, Jirong Wu, Åsmund Bjørnstad, Tatiana Belova, Susanne Dreisigacker, Etienne Duveiller, Pawan Singh

**Affiliations:** 1 International Maize and Wheat Improvement Center (CIMMYT), Texcoco, Edo. de México, Mexico; 2 Department of Plant Sciences, Norwegian University of Life Sciences, Ås, Norway; 3 Jiangsu Academy of Agricultural Sciences, Nanjing, China; Institute of Genetics and Developmental Biology, CHINA

## Abstract

Fusarium head blight (FHB) resistant line Soru#1 was hybridized with the German cultivar Naxos to generate 131 recombinant inbred lines for QTL mapping. The population was phenotyped for FHB and associated traits in spray inoculated experiments in El Batán (Mexico), spawn inoculated experiments in Ås (Norway) and point inoculated experiments in Nanjing (China), with two field trials at each location. Genotyping was performed with the Illumina iSelect 90K SNP wheat chip, along with a few SSR and STS markers. A major QTL for FHB after spray and spawn inoculation was detected on 2DLc, explaining 15–22% of the phenotypic variation in different experiments. This QTL remained significant after correction for days to heading (DH) and plant height (PH), while another QTL for FHB detected at the *Vrn-A1* locus on 5AL almost disappeared after correction for DH and PH. Minor QTL were detected on chromosomes 2AS, 2DL, 4AL, 4DS and 5DL. In point inoculated experiments, QTL on 2DS, 3AS, 4AL and 5AL were identified in single environments. The mechanism of resistance of Soru#1 to FHB was mainly of Type I for resistance to initial infection, conditioned by the major QTL on 2DLc and minor ones that often coincided with QTL for DH, PH and anther extrusion (AE). This indicates that phenological and morphological traits and flowering biology play important roles in resistance/escape of FHB. SNPs tightly linked to resistance QTL, particularly 2DLc, could be utilized in breeding programs to facilitate the transfer and selection of those QTL.

## Introduction

Fusarium head blight (FHB) is one of the most devastating diseases of wheat globally, leading to yield losses, quality degradation, and mycotoxin contamination, greatly threatening food and feed safety [1]. Various *Fusarium* species can cause this disease, but *F*. *graminearum* has the widest distribution and is considered to be the most potent one for being deoxynivalenol (DON) or nivalenol producer [2, 3]. DON affects animal and human health due to its toxic effects, and has also shown to be a virulence factor in FHB [4, 5]. In the European Union, legally enforceable DON thresholds were set up, with limits of 1.25 ppm in unprocessed wheat, 0.5 ppm in bread and biscuits, and 0.2 ppm in baby food [6].

Breeding for FHB resistance is a difficult task due to the complex inheritance and the strong genotype-by-environment interaction [7]. Hitherto, FHB resistance QTL have been reported on all the 21 wheat chromosomes; but few of them have been found to express consistently across different genetic backgrounds and environments [8, 9]. Even more, different resistance mechanisms have been identified, complicating further the development of FHB resistant wheat varieties. The first two mechanisms, also being the most widely acknowledged, were proposed by Schroeder and Christensen [10] and included Type I for initial infection and Type II for disease spread. Additionally, Miller and Arnison [11] and Mesterhazy [12] suggested three other mechanisms, i.e. Type III for toxin accumulation, Type IV for kernel infection, and Type V for yield reduction. Of these five mechanisms, Type II was regarded as the most effective and thus was extensively investigated; but the other components except Type V have been increasingly scrutinized in the last decade [8]. Different traits have been related to these FHB resistance mechanisms. For field spray and spawn inoculation, disease incidence scored at early stages (around 15 days post inoculation, dpi) was attributed to Type I resistance, whereas FHB severity or FHB index at later stages (25 dpi or later) was regarded as a combination of Type I and Type II resistance [13–15]. For point inoculation where the inoculum is directly injected into florets, only Type II resistance was measured [16]. Among the post-harvest traits, DON content and Fusarium damaged kernels (FDK) were related to Type III and Type IV resistance, respectively [8].

Numerous phenological and morphological traits have been shown to be associated with FHB resistance. Plant height (PH) and anther extrusion (AE) are the two parameters mostly investigated, and their negative correlation with Type I FHB susceptibility has been frequently documented [16–19]. Days to heading (DH) is often negatively correlated with Type I FHB susceptibility and usually resulting in disease escape [20]. Other traits include awned/awnless ears [12] and spike compactness [21].

Sources of resistance have been found in common wheat varieties mainly from Asia, Europe and the Americas, among which Sumai-3, Arina, and Frontana are the most known [9]. The International Maize and Wheat Improvement Center (CIMMYT) has also developed numerous FHB resistant breeding lines [22], among which Shanghai-3/Catbird (SHA3/CBRD) was characterized in a previous study and the results indicated a major QTL on 2DLc for Type I FHB resistance and several minor QTL, which were often associated with AE or PH [16]. In order to validate the resistance QTL and their association with morphological traits and flowering biology, another mapping population was developed using the resistant parent Soru#1 with Shanghai-3 (SHA3, a Chinese breeding line with high level of FHB resistance) in the pedigree and the susceptible parent Naxos as in Lu et al. [16].

## Materials and Methods

### Plant material

Soru#1 is a synthetic hexaploid wheat (SHW, i.e. the AABBDD genome is obtained after hybridizing durum wheat [AABB] and *Aegilops taushii* [DD]) derivative developed at CIMMYT with good FHB resistance [23]. It has a pedigree of SABUF/5/BCN/4/RABI//GS/CRA/3/*AE*.*SQUARROSA* (190), in which SABUF (pedigree SHA3//BUC/FLK) was anticipated to be the resistance source. In the study, Soru#1 was crossed with the German susceptible wheat cultivar ‘Naxos’ (Tordo/St.Mir808-Bastion//Miranet) to produce a recombinant inbred line (RIL) population with 131 F_6_ lines, using the single seed descent method.

### Field trials and phenotyping

The RIL population was evaluated in Mexico, Norway, and China, with two years of experiments at each site.

#### Mexico

The spray inoculated field experiments were conducted in the FHB nursery at the El Batán experimental station (altitude of 2,240 meters above sea level, coordinate 19.5°N, 98.8°W, with an average annual precipitation of 625 mm) of CIMMYT, Mexico, during the summer season (May to September). The population was planted in 2011 and 2012, with one and two replications, respectively, in 1-m double rows following a randomized complete block design. Each year, a mixture of 5 aggressive *F*. *graminearum* isolates were used for field inoculation, which comprised isolates 7, 12, 64, 158.2 and 702 in 2011 and 27, 32, 40, 44 and 158.2 in 2012. The isolates were collected from naturally infected wheat spikes in different places of the State of Mexico, following the protocols described in He et al. [24]. Spray inoculation was targeted to each line’s anthesis stage using an inoculum of 50,000 spores/ml and was repeated two days later. From anthesis to the early dough stage, the nursery was misted for 10 minutes each hour, from 9am to 8pm, to create a humid environment favourable for FHB development.

In 2011, FHB symptoms were scored three times at 20, 25, and 30 dpi, on 10 spikes that had been tagged at anthesis, whereas in 2012 the disease was scored only at 25 dpi. The total numbers of infected spikes and spikelets of each spike were counted to calculate the FHB index using the formula *FHB index* (%) = *Severity* x *Incidence* [25], where *Severity* is the averaged percentage of diseased spikelets, and *Incidence* is the percentage of symptomatic spikes. Plots were sickle harvested and threshed with a belt thresher set at low wind speed to retain scabby kernels. FDK rate was estimated only in 2012 by visually checking a random sample of kernels in a petri dish, where both scabby and shrivelled kernels were regarded as FDK. DON content, AE and tenacity of glumes (TG) were evaluated in both years. DON quantification was based on 2 g flour sampled from 20 g ground grain of each accession, using a Ridascreen Fast DON ELISA kit (R-Biopharm GmbH, Darmstadt, Germany) following the manufacturer’s instructions. AE was evaluated with a linear scale from 0 (no extrusion) to 9 (full extrusion) according to Skinnes et al. [17]. TG was scored during the milk to dough stages by manually separating glumes and kernels, using a scale from 1 (glumes can easily be separated) to 5 (glumes were usually torn when forcibly separated).

#### Norway

The spawn inoculated experiments were performed at the Vollebekk Research Farm (coordinate 59.7°N, 10.8°E) of the Norwegian University of Life Sciences, Ås, Norway, in 2011 and 2012. The population was planted in late April in hill plots of 40 x 50 cm in an alpha-lattice design with three replicates. Four locally collected isolates of *F*. *graminearum* with low to medium aggressiveness were used to colonize oat kernels to be used as spawn inoculum according to Skinnes et al. [17]. In 2011, the inoculum consisted of isolates 101118, 101018, 10177 and 101023, while isolates 101118, 101018, 200726 and 200838 were used in 2012. The infected oat kernels were distributed in the field at Zadoks’ GS 32–33. From then on, the field was mist irrigated for 10 minutes every hour from 7pm to 11pm every evening. The fungicide Zenit (Propiconazole + Fenpropidin) was applied prior to heading to prevent the natural infection of leaf and glume blotch caused by *Parastagonospora nodorum*. FHB evaluation was carried out at the beginning of maturity when a bundle of 10 spikes from the middle of each hill plot was counted for total and infected spikelets to calculate disease severity. For more detailed information on FHB screening at this location, refer to Lu et al. [16].

#### China

The point inoculated experiments were performed at the Jiangsu Academy of Agricultural Sciences (coordinate 32.0°N, 118.9°E), Nanjing, China, in the cropping seasons 2010–2011 and 2012–2013. The RIL population was sown in late October in 1.5 m rows at 33 cm distance in one randomized replication. A virulent *F*. *graminearum* strain F0603, isolated from a scabby wheat kernel in Nanjing, China, was used as inoculum in both experiments according to the protocols described in Shi et al. [26]. At the late heading stage before anthesis, around 15 spikes of each entry were inoculated at a single floret in the middle of the head, with 20 μl conidial suspension of 1 x 10^5^ spores/ml. At 20 dpi, FHB severity was scored for the inoculated spikes and the mean value of each entry was calculated for further analysis.

### Statistical analysis

The phenotypic data were analysed by the SAS program ver. 9.2. Analysis of variance (ANOVA) was carried out for each location with the PROC GLM module, and Pearson correlation coefficients were calculated using the PROC CORR function. The results of ANOVA were used for calculating the heritability estimates, using the formula *h*^*2*^ = $\sigma_{g}^{2}$/($\sigma_{g}^{2}$+$\sigma_{e}^{2}$/*r*) for single year and *h*^*2*^ = $\sigma_{g}^{2}$/($\sigma_{g}^{2}$+$\sigma_{g*y}^{2}$/*y* +$\sigma_{e}^{2}$/*ry*) for multiple years; in which $\sigma_{g}^{2}$ stands for genetic variance, $\sigma_{g*y}^{2}$ for genotype-by-year interaction, $\sigma_{e}^{2}$ for error variance, *y* for the number of years, and *r* for the number of replications [16].

### Genotyping

Genomic DNA was extracted from young leaves with the DNeasy Plant DNA extraction kit (QIAGEN). Genotyping was done with the Illumina iSelect 90K SNP wheat chip, containing 81,587 SNP markers [27]. Genotypes were called with the automatic clustering algorithm in Genome Studio V2011.1, followed by manual adjustment to recover polymorphic markers that were not correctly called by the algorithm. Additionally, two dwarfing genes *Rht-B1* and *Rht-D1* were genotyped using the Kompetitive Allele Specific PCR (KASP, LGC Limited, UK) based SNP markers developed at CIMMYT [28]. Using the same protocol, two SNPs identified in the current study to be associated with FHB resistance, Kukri_c36639_186 and Excalibur_c7282_512, were converted to KASP markers and used for genotyping the whole population. The *Vrn-A1* locus was genotyped with the primer pair VRN1AF/VRN1R described by Yan et al. [29]. Several SSR markers linked to known FHB resistance QTL [8, 16] were also investigated. Markers with missing data points less than 20% and segregation ratio within the range 0.5–2.0 were used for subsequent analysis.

### Linkage and QTL analysis

Linkage groups (LGs) were constructed using the program MST map [30] with a cut-off *p* value of 1e-6, maximum distance of 15 cM between markers, and minimum linkage group size of 2 cM. LGs were then assigned to chromosomes based on the BLASTN results of SNP sequences against chromosome survey sequences of A, B and D genomes [31, 32]. Only markers giving a hit to a single chromosome with ≥99% sequence identity and 100% coverage were assigned. Finally, the Maximum Likelihood algorithm in JoinMap v.4 was used to re-calculate the order and position of markers within each LG. QTL mapping was carried out with MapQTL v6.0 [33] using two mapping strategies. In the first strategy, interval mapping (IM) was performed to detect potential QTL for each trait, followed by multiple QTL mapping (MQM) for each QTL, using the closest linked markers to each QTL detected in IM as cofactors. In the second strategy, the same mapping algorithms were adopted except for the use of DH and PH as covariates, this could be helpful in the identification of QTL independent of or masked by the two traits. A significant QTL was defined in this study as with a LOD score higher than 3.0; minor QTL with LOD values higher than 2.0 were also reported if they were significant in at least one environment. LGs and LOD curves were drawn by the software MapChart ver. 2.3 [34].

## Results

### Phenotypic analysis

**Fig 1:**
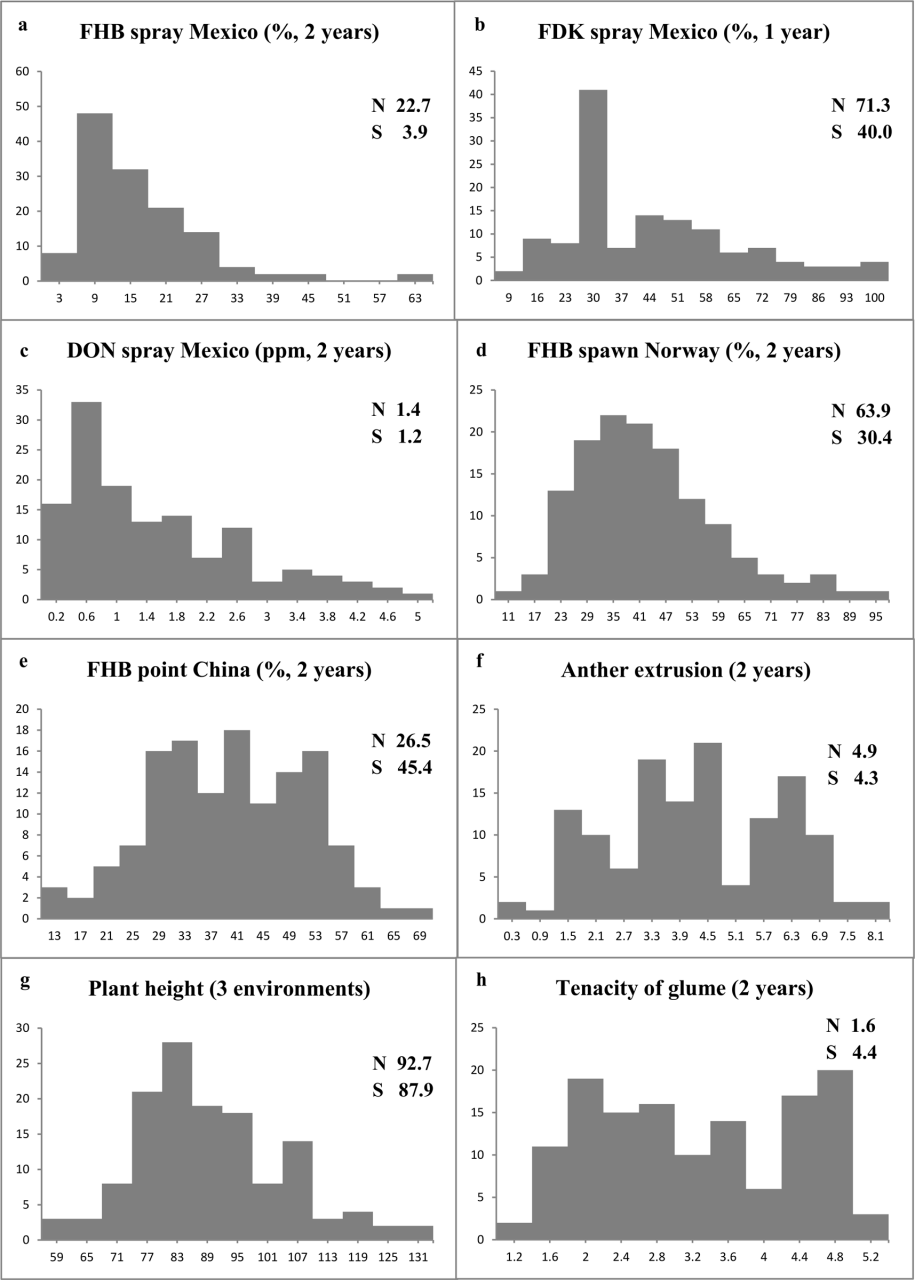
Frequency distribution of FHB traits in the Soru#1 x Naxos population based on the mean data across environments. *N* Naxos, *S* Soru#1.

In terms of spray or spawn inoculation, the disease severity in the field in Mexico in both years (with grand mean value of 13.4%) was lower than in Norway (39.7%). Continuous distribution patterns were found at both locations (Fig 1A–1D), indicating quantitative inheritance of FHB resistance. Transgressive segregations were observed at both high and low disease levels, and the resistant parent Soru#1 always exhibited a much lower infection than the susceptible parent Naxos except for DON content, where both parents showed relatively low values (Fig 1C). Regarding the point inoculated experiments in China, contrasting results were obtained in 2011 and 2013, with the former showing very low FHB severity with a grand mean of 12.8% and the latter exhibiting extreme infection with a grand mean of 63.1%. Unlike in spray and spawn experiments, Naxos showed much lower infection than Soru#1 after point inoculation (Fig 1E).

Significant genotypic effects were detected among the RILs for all the traits, as well as the genotype x year effects, although their significance levels differed from each other (Table 1). Moderate to high heritability estimates were obtained for FHB traits in spray and spawn inoculation, but a low value of 0.34 was observed for point inoculation, possibly due to the big differences in disease pressure in the two years (Table 1). Correlations across locations/years among the FHB parameters from spray/spawn experiments were all highly significant (Table 2). Since in 2011 the FHB index at 25 dpi and the area under the disease progress curve (AUDPC) showed a very high correlation of *r* = 0.99, only the former was used in this study. The two point inoculated experiments showed only a marginal correlation of *r* = 0.34; the correlation between spray/spawn and point inoculated experiments was usually very low and non-significant (Table 2). Regarding the phenological and morphological traits, only PH consistently showed a moderate correlation with FHB traits. The correlation of FHB with DH and TG was usually low, and that between FHB and AE was also low and often non-significant in spray inoculated experiments; but the predicted correlation for FHB vs. AE in spawn inoculated experiments was higher (Table 3).

**Table 1:** Analysis of variance for Fusarium head blight and associated traits and their heritability estimates in the Soru#1 x Naxos population.

Traits	Source	DF	Mean square	*F* value	*P* value	Heritability
FHB spray	Genotype	132	298.20	2.80	<0.0001	0.65
	Year	1	4184.37	39.45	<0.0001	
	Genotype x Year	130	106.06	7.53	<0.0001	
	Rep (Year)	1	39.73	2.82	0.0954	
	Error	130	14.08			
FDK spray	Genotype	134	918.52	8.75	<0.0001	0.89
	Rep	1	3209.69	30.57	<0.0001	
	Error	128	105.00			
DON spray	Genotype	131	3.32	2.34	<0.0001	0.57
	Year	1	0.67	0.47	0.4943	
	Genotype x Year	117	1.42	1.87	0.0003	
	Rep (Year)	1	0.14	0.18	0.6735	
	Error	127	0.76			
FHB spawn	Genotype	132	1611.98	6.60	<0.0001	0.85
	Year	1	237338.23	972.06	<0.0001	
	Genotype x Year	132	244.16	1.89	<0.0001	
	Rep (Year)	4	1393.16	10.80	<0.0001	
	Error	526	129.05			
FHB point	Genotype	132	280.81	1.53	0.0079	0.34
	Year	1	169469.48	920.42	<0.0001	
	Error	132	184.12			
Anther extrusion	Genotype	132	8.55	3.94	<0.0001	0.74
	Year	1	0.52	0.45	0.5035	
	Genotype x Year	132	2.17	0.28	0.0002	
	Rep (Year)	1	0.45	0.39	0.5308	
	Error	132	1.15			
Plant height	Genotype	132	1033.62	14.76	<0.0001	0.94
	Year	2	48328.35	690.21	<0.0001	
	Genotype x Year	264	70.02	5.17	<0.0001	
	Rep (Year)	3	34.13	2.52	0.0575	
	Error	394	13.53			
Tenacity of glumes	Genotype	132	3.17	5.98	<0.0001	0.83
	Year	1	2.65	5.00	0.0270	
	Genotype x Year	132	0.53	2.45	<0.0001	
	Rep (Year)	1	2.54	11.79	0.0008	
	Error	132	0.22			

**Table 2:** Pearson correlation coefficients among FHB traits in the Soru#1 x Naxos population.

	Spray					Spawn		Point	
	FHB11	DON11	FHB12	FDK12	DON12	FHB11	FHB12	FHB11	FHB13
Spray									
FHB11	1								
DON11	0.64**	1							
FHB12	0.54**	0.39**	1						
FDK12	0.39**	0.35**	0.49**	1					
DON12	0.46**	0.41**	0.62**	0.42**	1				
Spawn									
FHB11	0.62**	0.57**	0.65**	0.43**	0.52**	1			
FHB12	0.55**	0.55**	0.57**	0.47**	0.59**	0.74**	1		
Point									
FHB11	0.26*	0.16	0.20	0.10	0.23	0.23	0.27*	1	
FHB13	0.18	0.16	0.05	0.01	0.28*	0.07	0.18	0.34**	1

* *P*<0.01

** *P*<0.0001

**Table 3:** Pearson correlation coefficients between FHB parameters and associated traits in the Soru#1 x Naxos population.

	Days to heading	Anther extrusion	Tenacity of glumes	Plant height
Spray				
FHB11	-0.33**	-0.14	-0.29*	-0.47**
FHB12	-0.11	-0.34**	-0.03	-0.45**
FHB mean	-0.34**	-0.17	-0.28*	-0.56**
FDK12	0.11	-0.17	-0.41**	-0.61**
DON11	-0.28*	-0.12	-0.24*	-0.36**
DON12	-0.39**	-0.25*	-0.01	-0.40**
DON mean	-0.44**	-0.20*	-0.10	-0.44**
Spawn				
FHB11	-0.29*	(-0.37**)	(-0.04)	-0.49**
FHB12	-0.44**	(-0.38**)	(0.00)	(-0.56**)
FHB mean	-0.40**	(-0.40**)	(-0.02)	-0.57**

* *P*<0.01

** *P*<0.0001

The associated traits were measured in the same field trials as FHB parameters, except for anther extrusion, tenacity of glumes, and plant height 2012 which were unavailable in spawn inoculated experiments, in those cases, mean values in spray inoculated experiments (the former two) and the values in 2011 from the same location (the latter one) were used and the results were presented in parentheses.

### Linkage analysis

In total, 10,372 high-quality SNP markers were scored for this population, of which 10,255 SNPs along with 16 SSRs, *Vrn-A1* and *Rht-D1* were allocated to 40 LGs representing all the 21 chromosomes. *Rht-B1* was monomorphic in this population (*Rht-B1a*) and was not scored. The remaining markers were either unlinked or found in small LGs (usually smaller than 5cM) and thus were discarded. A refinement process was carried out subsequently to select only one marker from each cluster of co-segregating SNPs, resulting in a linkage map with 3,267 markers, covering 3,216 cM and having a density of about 1 marker/cM. A and B genome chromosomes were well covered with more than 200 SNPs each, except 4A, 5B and 6B harbouring 100–150 SNPs. In contrast, D genome chromosomes were generally poorly covered, having from 107 markers for 2D to only 10 for 4D.

### QTL mapping for FHB traits

Resistance to FHB in this population was controlled by two major QTL on 2DLc and 5AL (at *Vrn-A1*), and several minor QTL, mainly from Soru#1 with a few from Naxos (Tables 4, 5 and 6)

**Table 4:** QTL for FHB traits after spray and spawn inoculations in the Soru#1 x Naxos population and their association with other traits.

QTL	Position	Left marker	Right marker	Spray inoculation							Spawn inoculation			R source	Associated traits
				FHB index			FDK	DON content			FHB severity				
				2011	2012	Mean	2012	2011	2012	Mean	2011	2012	Mean		
2DLc	55.3–58.2	Kukri_c36639_186	Ex_c7282_512	**18.1**	**15.8**	**15.9**	7.4	**20.1**	**13.6**	**16.5**	**15**	**22.4**	**18.2**	S	
2DL	162.6–163.4	BS00021881_51	GENEU3588_374	6.3	**8.6**	4.4		**9.4**	**7.5**	**6.1**		5.3	3.2	N	AE
4AL	101.2–102.6	Ex_c11968_204	RAC875_c35979_263	7.1		5.4					**6.8**	4	5.9	S	DH
4DS	0.0–15.9	Rht-D1	D_c56766_278	** **		** **	**19**				** **			N	PH
5AL	167.2–171.2	Vrn-A1	Ex_c31769_793	**36.8**	**11.2**	**22.8**	**5.7**	**17.5**	**14.1**	**17.6**	**8**	**7.5**	**7.8**	S	DH, PH
Accumulated percentage of variation explained				72.9	40.1	51.7	32.1	52.4	39.9	40.2	35.7	49.9	44.4		

The percentage of explained phenotypic variation in the multiple regression models is shown

QTL are listed if they were over the LOD threshold of 3 (in bold) in at least one environment

*N* Naxos, *S* Soru#1, *AE* anther extrusion, *PH* plant height, *DH* days to heading

**Table 5:** QTL for FHB traits after spray and spawn inoculations, with days to heading and plant height as covariates.

QTL	Position	Left marker	Right marker	Spray inoculation							Spawn inoculation			R source
				FHB index			FDK	DON content			FHB severity			
				2011	2012	Mean	2012	2011	2012	Mean	2011	2012	Mean	
2AS	7.2–8.6	Ex_c18324_390	BS00022331_51		4.5						**5.9**	4.3	5.2	N
2DLc	53.2–57.8	w_Ku_c8712_14751858	GENEU0808_728	**9.7**	**15.9**	**15.2**	**4.7**	**14.2**	**17.1**	**12.9**	**8.7**	**13.4**	**12.2**	S
2DL	162.6–164.4	BS00021881_51	Kukri_c31995_1948		**8.2**	3.4		5.5	**6.5**	**5.6**		**5.7**	**4.7**	N
4DS	0–15.9	Rht_D1	D_c56766_278	**4.6**		3.2		5.4						S
5AL	173.4–175.2	Ku_c12469_983	IAAV4799	**6.8**		**4.5**		**6**		**4.3**				S
5DL	5.8–10.6	Ra_c27043_437	IAAV2323						4.7	2.7		**6.4**	3.8	S
Accumulated percentage of variation explained				21.1	28.6	26.3	4.7	31.1	28.3	25.5	14.6	29.8	25.9	

The percentage of explained phenotypic variation in the multiple regression models is shown

QTL are listed if they were over the LOD threshold of 3 (in bold) in at least one environment

*N* Naxos, *S* Soru#1

**Table 6:** QTL for FHB severity after point inoculation in the Soru#1 x Naxos population.

QTL	Position	Left marker	Right marker	Point inoculation			R source
				2011	2013	Mean	
2DS	16.0–22.0	D_F1BEJMU02GB94Z_188	wExc1408_2704613	**13.5**			N
3AS	12.5–18.0	Kukri_c96747_274	wC11_r_c4157_1965583		**9.9**		S
4AL	101.2–102.6	Ex_c11968_204	RAC875_c35979_263	6.6			S
5AL	167.2–167.6	Vrn-A1	Ex_c7729_144		**12.3**	**10.9**	S
Accumulated percentage of variation explained				20.1	22.2	10.9	

The percentage of explained phenotypic variation in the multiple regression models is shown

QTL are listed if they were over the LOD threshold of 3 (in bold) in at least one environment

*N* Naxos, *S* Soru#1

In the spray inoculated experiments, QTL on 2DLc and 5AL were the most prominent, contributing to all three FHB resistance components and being significant in both years (Table 4). The former explained 14–20% of the phenotypic variation for FHB and DON, while the latter accounted for 11–37% of the phenotypic variation for the two traits; both showed much lower phenotypic effects of up to 7% for FDK. Additionally, a QTL on 2DL derived from Naxos was also consistently significant for FHB and DON, explaining phenotypic variation of 6–9%. A QTL for FDK was mapped at the *Rht-D1* locus on 4DS, accounting for 19% of the phenotypic variation; but it had no direct effect on FHB and DON. Additional minor QTL on 4AL was identified only in 2011 (Table 4).

**Fig 2:**
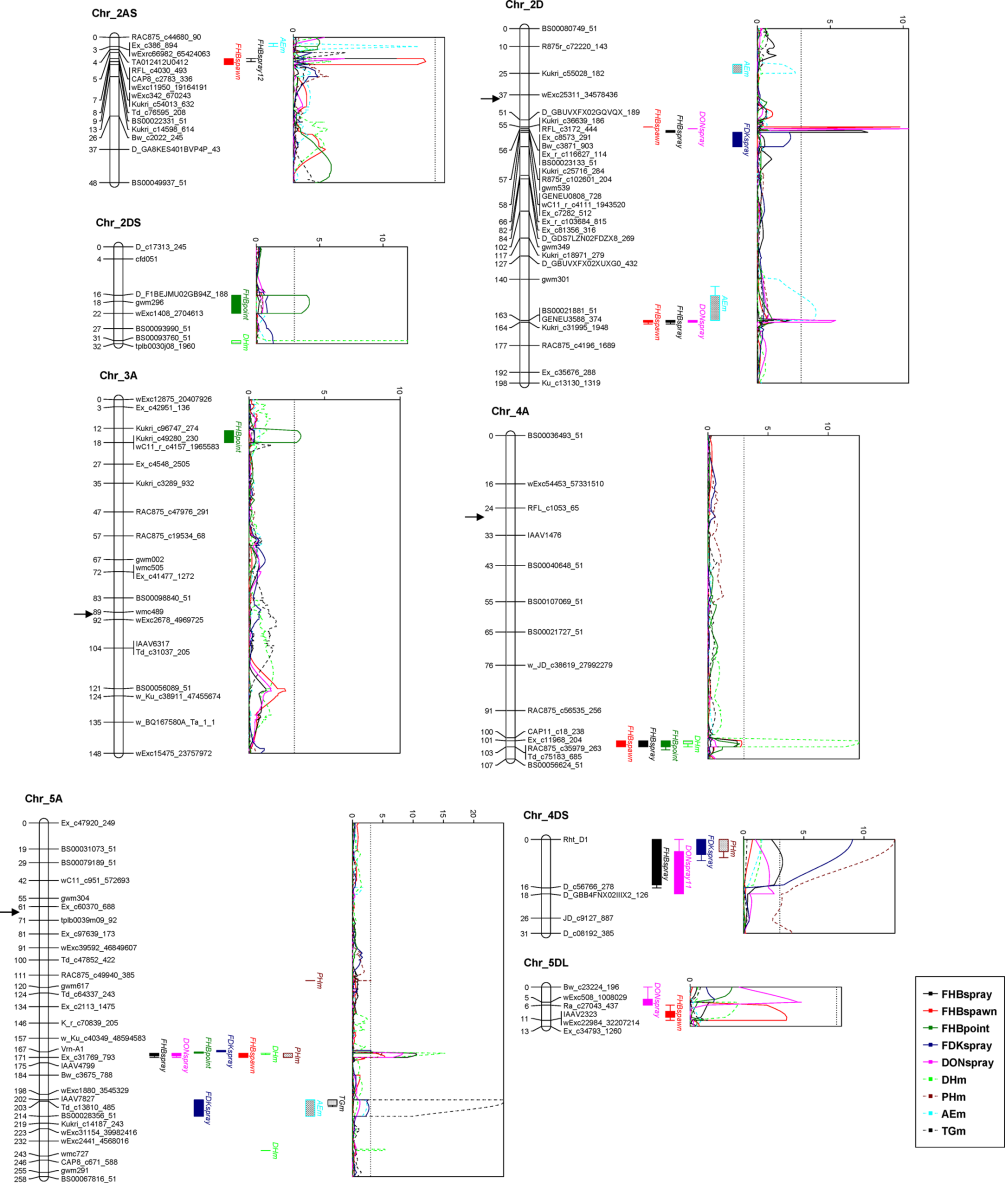
QTL for FHB parameters and their associated traits based on mean phenotypic data. If there was no QTL detected based on the mean, the environment with significant QTL effect was marked instead, with the year behind the QTL name. Genetic distances are shown in centimorgans to the left of the chromosomes. A threshold of 3.0 is indicated by a dashed vertical line in the LOD graphs. Only framework markers are presented except for the QTL regions. The approximate positions of centromeres are indicated by arrows.

In the spawn inoculated experiments, QTL on 2DLc, 2DL, 4AL and 5AL were identified too. Compared to those reported in spray experiments, the ones for spawn inoculation were mapped to very similar locations and showed similar phenotypic effects, except for the one on 5AL with lower impact on FHB (Table 4, Fig 2).

When PH and DH were used as covariates, QTL mapping results differed greatly with those reported above (Table 5). The QTL on 4AL and 4DS disappeared, and that on 5AL at *Vrn-A1* was significant only in 2011 for FHB and DON, with much lower phenotypic effects. The ones on 2DLc and 2DL remained significant but their phenotypic contributions were generally decreased. Interestingly, three new putative QTL were detected with this strategy, being localized on 2AS, 4DS and 5DL (Table 5). The one on 4DS was mapped to the same marker interval as that for FDK at *Rht-D1*, but its resistance came from Soru#1 instead of Naxos, implying a QTL independent of the height reduction effect of *Rht-D1*, which was also supported by the LOD curve with a peak proximal to *Rht-D1* (Fig 2).

In the point inoculated experiments, no QTL stably expressed in both years was detected. In 2011, QTL on 2DS and 4AL were detected, with phenotypic contributions of 14% and 7%, respectively; whereas in 2013, QTL on 3AS and 5AL were identified, explaining phenotypic variations of 10% and 12%, respectively (Table 6). Soru#1 contributed the resistant allele for all but the 2DS QTL. It is noteworthy that the QTL on 4AL and 5AL were mapped to the same regions as those in spray and spawn experiments.

### QTL mapping for AE, TG, PH and DH

For AE, five QTL were detected on chromosomes 2AS, 2DS, 2DL, 4AL and 5AL, explaining phenotypic variations of 6–14% (Table 7). Both parents contributed to high AE; but the contribution of Naxos was more important. For TG, only one QTL contributed by Soru#1 was found on 5AL over the two years (Fig 2), explaining the phenotypic variation of 48% and 58%, respectively.

**Table 7:** QTL for anther extrusion in the Soru#1 x Naxos population and their association with FHB traits.

QTL	Position	Left marker	Right marker	2011	2012	Mean	High AE	Association
2AS	2.9–8.6	Ex_c386_894	BS00022331_51	6.9	**10.6**	8.9	N	FHBs
2DS	19.6–25.3	BS00021912_51	Kukri_c55028_182	6.3	5.5	6.3	S	
2DL	139.6–162.6	gwm301	BS00021881_51	5.8	**13.5**	**11.5**	N	FHBs, DON
4AL.2	24.7–25.1	R875r_c117027_137	IAAV3545		**8.2**	6.5	S	
5AL	201.6–213.8	IAAV7827	BS00028356_51	9.2	** **	7	N	FDK
Accumulated percentage of variation explained				28.2	37.8	40.2		

The percentage of explained phenotypic variation in the multiple regression models is shown

QTL with LOD values higher than 2 are listed, and those with LOD higher than 3 are bolded

*N* Naxos, *S* Soru#1, *FHBs* FHB index or severity after spray or spawn inoculation

For PH, the QTL at *Rht-D1* was the most important, contributing to around 30% of the phenotypic variation, followed by the one at *Vrn-A1* on distal 5AL (with phenotypic effects of 13–18%) and the one on proximal 5AL (6–8%) (S1 Table and Fig 1). Soru#1 contributed to tall statue at the two loci on 5AL and Naxos did so at *Rht-D1*. For DH, QTL were often exclusively found in either Mexico or Norway, except the shared one at *Vrn-A1*. The two QTL unique to Mexico were on 2BS and 2DS, possibly at *Ppd-B1* and *Ppd-D1*, respectively, while the ones unique to Norway were on 4AL and distal 5AL (S2 Table). Naxos contributed to delayed heading for all loci but *Vrn-A1*.

### Coincidence or linkage of QTL between FHB and associated traits

Coincidence or linkage of QTL for different traits was frequently observed in this study (Fig 2). The most prominent locus was *Vrn-A1*, where QTL for all the FHB traits, PH and DH coincided, and QTL for TG, AE and FDK were found in a distal site of approx. 30 cM. On the distal region of 4AL, a QTL for DH coincided with those for FHB. At *Rht-D1* on 4DS, QTL for PH and FDK coincided and were in close linkage with those for FHB and DON. The QTL for AE on 2AS and 2DL were either closely linked to or coincided with those for FHB and DON, whereas the one on 5AL showed its association with only FDK but was linked to the major QTL for FHB traits in its proximal direction with a genetic distance of approx. 30 cM, and this kind of linkage was also found for the 2DS QTL.

### Development of KASP markers for the 2DLc QTL

The two SNPs flanking the 2DLc QTL were converted to KASP markers (S3 Table), and the genotyping results were identical with those from Illumina iSelect 90K chip, demonstrating the reliability of the KASP protocol. For both Kukri_c36639_186 and Excalibur_c7282_512, the two KASP alleles can be clearly differentiated in the Soru#1 x Naxos population (S1 Fig). Further genotyping of the two KASPs on an association mapping panel of Norwegian wheat varieties revealed, however, that Excalibur_c7282_512 generated a complicated pattern with three main clusters, in contrast to Kukri_c36639_186 with two main clusters (S2 Fig).

## Discussion

Phenological and morphological traits like DH and PH are often reported to be associated with field resistance to FHB [18, 20], which was also true for the current study. As proposed by Lu and Lillemo [35], two strategies could be utilized to distinguish true resistance QTL from those caused by confounding effects of DH and PH, i.e. 1) analysing all the traits separately and checking the coincidence of QTL for disease and associated traits to single out true disease resistance QTL, and 2) using adjusted disease data for which the confounding effects had been excluded. In the current study, both methods were adopted for the successful identification of true FHB resistance QTL and the evaluation of the confounding factors at QTL level. Using these methods, it was evident that the influence of DH and PH on FHB was mainly through the QTL on 5AL at *Vrn-A1* and that on 4AL, and their contribution to FHB substantially decreased or disappeared when DH and PH were used as covariates. The utilization of the second method showed an additional advantage in the identification of putative minor QTL that would have not been found with the traditional methods, e.g. the QTL on 2AS, 4DS and 5DL (Table 5).

As expected, many QTL were mapped to similar regions as those in the SHA3/CBRD x Naxos population [16] based on shared SSR markers (2DLc, 2DL and 2DS) or inferred from chromosomal location, resistance source and associated traits (2AS, 4AL and 5AL). Considering that both SHA3/CBRD and Soru#1 have Shanghai-3 in their pedigree, the shared QTL might be inherited from Shanghai-3, i.e. 2DLc, 4AL and 5AL.

Like in Lu et al. [16], the 2DLc QTL was the most important ‘true QTL’ in the present study. In Lu et al. [16] it coincided with AE and PH, but in the present work it was independent of those traits. This QTL was initially identified in a Chinese line Wuhan-1 [36] and was subsequently confirmed in Chinese germplasm like Wangshuibai [37, 38] and CJ9306 [39, 40], as well as in the CIMMYT wheat line Mayoor [41, 42], in the Swiss winter wheat Arina [43] and in the US winter wheat line VA00W-38 [44]. This QTL was regarded as a major QTL in most of the aforementioned studies and expressed stably across environments, explaining 10–30% of phenotypic variations; but in Paillard et al. [43] it showed small effects and was identified in only one out of six environments. It is interesting to note the Type I vs. Type II resistance of this QTL in different genetic backgrounds. In Wuhan-1, it conferred exclusively Type II resistance [36]; but in Wangshuibai it contributed only to Type I resistance [37] and was non-significant in Type II experiments [45]. In CJ9306, only point inoculated experiments were performed and it was consistently significant for Type II resistance [40]. In Mayoor, its contribution to Type II resistance was confirmed [42] and it may also confer Type I resistance in a follow-up study [41]. In Lu et al. [16] this QTL was associated mainly with Type I resistance and showed minor effects on Type II resistance; whereas in the current study it exhibited only Type I resistance. In the remaining studies, the two mechanisms could not be separated; but it is already very clear that this QTL contributes to both Type I and Type II resistance and that the relative importance of the two components vary across genetic backgrounds and environments. Pathological experiments provided further evidence for the function of this QTL. In a recent research by Long et al. [46], visual and microscopic observations were carried out in two pairs of sister lines differing at the 2DLc QTL from Wuhan-1, and the results demonstrated that the QTL suppressed fungal infection in inoculated spikelets and reduced spread from spikelets to rachis. Our previous results indicated the frequent occurrence of this QTL in CIMMYT germplasm [22, 24], most likely due to the wide use of Wuhan-1 and Shanghai-3 in the breeding programs.

The 5AL chromosome region showed effect on all the traits evaluated in the present study. The QTL for FHB parameters was likely to be conferred by the pleiotropic effects of *Vrn-A1*, for which similar results were reported by Gervais et al. [47] and Klahr et al. [48]. Closely linked to this QTL was a locus for TG, FDK and AE (Fig 2), likely to be the *Q* locus due to its significant effect on TG. The high collinearity of QTL in this region (centromere ~ *QTL*_PH_ ~ *Vrn-A1* ~ *Q*) between our results and those of Kato et al. [49] provided additional evidence for the identity of the *Q* locus. *Q* is a key factor in the evolution and cultivation of domesticated wheat, due to its role in conditioning the free-threshing habit. Additionally, it pleiotropically affects many important traits such as plant height, spike shape, ear compactness [50]. The identification of QTL for FDK and AE at *Q* may simply be a consequence of TG, i.e. high TG reduced glume opening, which subsequently led to low AE and FDK. It was unexpected to map *Q* in this study, since both common and durum wheat (the latter being a parental species of Soru#1, a SHW derivative) are expected to have the free-threshing *Q* allele at this locus, rather than the non-free-threshing *q* allele found in *T*. *dicoccoides*, *T*. *dicoccum* etc. A possible reason is that different *Q* alleles are present in durum vs. common wheat, which was supported by results of Jantasuriyarat et al. [51] and Faris [50]. Like in Buerstmayr et al. [21], this locus also contributed to spike compactness in the current study, explaining 23% phenotypic variation (data no shown); but it was not associated with strong FHB resistance as in Buerstmayr et al. [21], Chu et al. [52], and Zhang et al. [53].

Compared to the 2DLc QTL, the one on the distal region of 2DL was little known. This QTL was identified in Lu et al. [16] for FHB severity in both spray and spawn experiments, with low phenotypic effects of merely 2–3%. In the present study, it explained more phenotypic variation for FHB resistance after spray and spawn inoculation (5–9%), and also contributed to DON resistance (8–9%). Taken together, these results exhibited the validity of this QTL for Type I and III resistance. Another report of this QTL was Lin et al. [37], where it was detected for Type I resistance in Wangshuibai in only one environment. Recently, Diethelm et al. [54] identified a *NPR1*-like gene and mapped it to the homoeologous regions of 2AL and 2DL chromosomes. Although map comparison indicated that the 2DL *NPR1*-like gene falls into the 2DL QTL region identified in the current study, the diagnostic marker for the former was monomorphic in the Soru#1 x Naxos population (all lines have the resistant allele, data not shown), implying that *NPR1-*like gene is not the underlying gene for this QTL.

It has been documented that *Rht-D1b* was associated with Type I susceptibility to FHB, with possible underlying mechanisms being disease escape, pleiotropy or tight linkage [18]. In our study, however, it showed negative impact only on FDK; and surprisingly it was associated with reduced FHB index and DON content in 2011 in Mexico under spray inoculated experiment. With close scrutiny of the LOD profile, it can be observed that the peak regions of QTL for the two latter traits were proximal to *Rht-D1*, implying a resistance QTL in close linkage with *Rht-D1*. Indeed, the QTL can only be detected when PH and DH were used as covariates, otherwise its effects will be masked by *Rht-D1*. Similarly, Srinivasachary et al. [55] also found FHB QTL peaks in a short distance away from the *Rht-D1* locus; but in their study the dwarfing allele *Rht-D1b* was in repulsive linkage with FHB resistance, whereas in our study the linkage was in coupling, which was very favourable for breeders aiming at short FHB resistant varieties. Nevertheless, phenotypic effects of this QTL in our study were too small to make any solid conclusion, let alone the evident pleiotropic effect of *Rht-D1* on FDK.

Most of other minor QTL can be assigned to known QTL clusters as reported in Liu et al. [8], based on either shared SSR markers or the comparison with Lu et al. [16]. QTL for Type II resistance were not very robust in this study, probably due to a lack of strong resistance to fungal spread in both parents.

Five QTL were identified for AE, yet the accumulated phenotypic variation explained was only around 30%, indicating a typical quantitative trait that is in accordance with previous reports [16, 17, 56]. It is noteworthy that the populations in Skinnes et al. [17] and Buerstmayr and Buerstmayr [56] shared a common parent Arina, and those in Lu et al. [16] and ours shared a common parent Naxos; nevertheless, few QTL was found in common among these studies, which was very unexpected considering that a total of 18 QTL have been reported. The QTL on 7AL found by Skinnes et al. [17] and Lu et al. [16] may be the same, and the QTL found on 4AL in this study could be the same as reported by Buerstmayr and Buerstmayr [56], but evidence in the form of shared markers is lacking. Like in those studies, QTL for FHB and AE were often overlapped or linked in our study. Yet the phenotypic correlation was generally low in spray inoculated experiments which could have been largely compromised by the repulsive linkage of QTL for the two traits on 5AL; whereas in spawn inoculated experiments the correlation increased due to the weaker effects of *Vrn-A1* that attenuated the repulsive linkage.

The main purposes of QTL mapping are the identification of closely linked markers for MAS and laying foundations for fine mapping and cloning of underlying resistance genes. With the advent of next-generation sequencing technology, a high quantity of SNP markers have been developed and utilized in genetic studies. The Illumina iSelect 90K wheat chip [27] represents a rich collection of wheat SNP markers that is being used worldwide, providing great opportunities for QTL mapping and genome-wide association studies. Benefited from the high-density of the SNPs, the resolution of the current linkage map was much improved compared with the one in Lu et al. [16], and the QTL were mapped at higher precision. The two SNPs flanking the 2DLc QTL, Kukri_c36639_186 and Excalibur_c7282_512, were converted to KASP markers and the results were consistent with those obtained from the Illumina iSelect 90K chip, demonstrating the reliability of the KASP protocol; nevertheless, the latter showed complicated cluster patterns in an association mapping panel, implying cross amplification from genomic regions other than 2DLc. Indeed, blast search indicated that Excalibur_c7282_512 mapped to both 2AL and 2DL, making it less suitable for MAS than Kukri_c36639_186. For those researchers/breeders not having access to SNP based genotyping platforms, the SSR marker *gwm539* can be used instead. This marker has been shown to be tightly linked to the 2DLc QTL in numerous studies [16, 36–40, 42, 43], as well as in the current study (Fig 2).

## Supporting Information

S1 Fig KASP profiles for Kukri_c36639_186 (a) and Excalibur_c7282_512 (b), the two SNPs flanking the 2DLc QTL, in the Soru#1 x Naxos population. *Orange* dots stands for the female parent Soru #1, *light blue* for the male parent Naxos, *red* for progenies with the Soru #1 allele, *blue* for progenies with the Naxos allele, *green* for heterozygous, *yellow* for progenies with poor calling quality, *black* for negative control, and *purple* for failed calling.
(DOCX)

S2 Fig KASP profiles for Kukri_c36639_186 (a) and Excalibur_c7282_512 (b), the two SNPs flanking the 2DLc QTL, in an association mapping panel of Norwegian wheat varieties. *Red* dots stand for progenies with the Soru #1 allele, *blue* for progenies with the Naxos allele, *gray* for negative control, and *black* for failed calling.
(DOCX)

S1 Table QTL for plant height in spray and spawn experiments. (DOCX)

S2 Table QTL for days to heading in spray and spawn experiments. (DOCX)

S3 Table Sequence information of the two KASP markers for SNPs flanking the 2DLc QTL. (DOCX)
