# Supplementary material for: QTL Characterization of Fusarium Head Blight Resistance in CIMMYT Bread Wheat Line Soru#1
Source: PLoS One. 2016 Jun 28;11(6):e0158052. doi: 10.1371/journal.pone.0158052 (PMC4924825; doi:10.1371/journal.pone.0158052)
Supplement: S1 Fig — KASP profiles for Kukri_c36639_186 (a) and Excalibur_c7282_512 (b), the two SNPs flanking the 2DLc QTL, in the Soru#1 x Naxos population. Orange dots stands for the female parent Soru #1, light blue for the male parent Naxos, red for progenies with the Soru #1 allele, blue for progenies with the Naxos allele, green for heterozygous, yellow for progenies with poor calling quality, black for negative control, and purple for failed calling. (DOCX) [file pone.0158052.s001.docx]

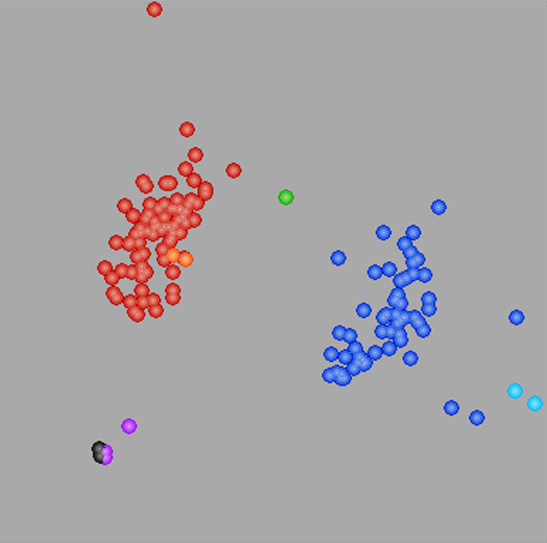

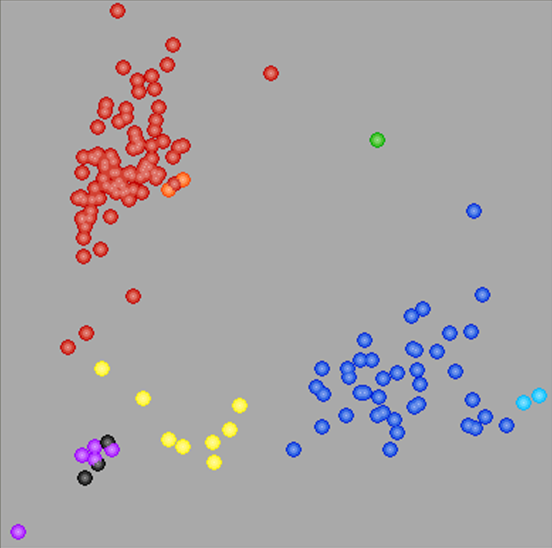


**b**

**a**

**S1 Fig** KASP profiles for Kukri_c36639_186 (a) and Excalibur_c7282_512 (b), the two SNPs flanking the 2DLc QTL, in the Soru#1 x Naxos population. *Orange* dots stands for the female parent Soru #1, *light blue* for the male parent Naxos, *red* for progenies with the Soru #1 allele, *blue* for progenies with the Naxos allele, *green* for heterozygous, *yellow* for progenies with poor calling quality, *black* for negative control, and *purple* for failed calling
