# Supplementary material for: QTL Characterization of Fusarium Head Blight Resistance in CIMMYT Bread Wheat Line Soru#1
Source: PLoS One. 2016 Jun 28;11(6):e0158052. doi: 10.1371/journal.pone.0158052 (PMC4924825; doi:10.1371/journal.pone.0158052)
Supplement: S2 Fig — KASP profiles for Kukri_c36639_186 (a) and Excalibur_c7282_512 (b), the two SNPs flanking the 2DLc QTL, in an association mapping panel of Norwegian wheat varieties. Red dots stand for progenies with the Soru #1 allele, blue for progenies with the Naxos allele, gray for negative control, and black for failed calling. (DOCX) [file pone.0158052.s002.docx]

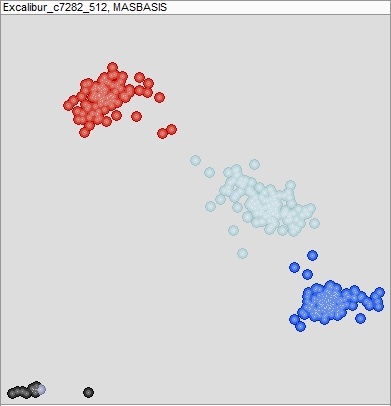

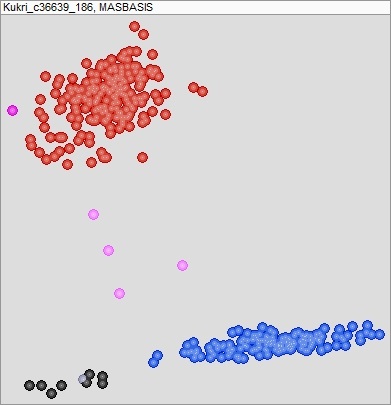


**b**

**a**

**S2 Fig** KASP profiles for Kukri_c36639_186 (a) and Excalibur_c7282_512 (b), the two SNPs flanking the 2DLc QTL, in an association mapping panel of Norwegian wheat varieties. *Red* dots stand for progenies with the Soru #1 allele, *blue* for progenies with the Naxos allele, *gray* for negative control, and *black* for failed calling
