# Supplementary material for: QTL Characterization of Fusarium Head Blight Resistance in CIMMYT Bread Wheat Line Soru#1
Source: PLoS One. 2016 Jun 28;11(6):e0158052. doi: 10.1371/journal.pone.0158052 (PMC4924825; doi:10.1371/journal.pone.0158052)
Supplement: S1 Table — (DOCX) [file pone.0158052.s003.docx]

**S1** **Table** QTL for plant height in spray and spawn experiments

| **QTL** | **Position** | **Left marker** | **Right marker** | **Spray inoculation** | | |  | **Spawn inoculation** | **High PH** | **Association** |
| --- | --- | --- | --- | --- | --- | --- | --- | --- | --- | --- |
|  |  |  |  | **2011** | **2012** | **Mean** |  | **2011** |  |  |
| 4DS | 0.0-15.9 | Rht-D1 | D_c56766_278 | 29.9 | 31.1 | 29.0 |  | 38.8 | N | FDK |
| 5AL.1 | 116.5-118.3 | Ra_c6930_1753 | BS00023076_51 | 5.8 | 8.3 | 8.3 |  |  | S |  |
| 5AL.2 | 167.2-170.8 | Vrn-A1 | w_Ku_c6977_12078885 | 18.1 | 12.8 | 13.6 |  | 13.2 | S | FHBs, FHBp, FDK, DON |
| Accumulated percentage of variation explained | | | | 53.8 | 52.2 | 50.9 |  | 52.0 |  |  |

The percentage of explained phenotypic variation in the multiple regression models is shown

QTLs with LOD values higher than 3 are listed

*N* Naxos, *S* Soru#1, *FHBs* FHB index or severity after spray or spawn inoculation, *FHBp* FHB severity after point inoculation
