# Supplementary material for: QTL Characterization of Fusarium Head Blight Resistance in CIMMYT Bread Wheat Line Soru#1
Source: PLoS One. 2016 Jun 28;11(6):e0158052. doi: 10.1371/journal.pone.0158052 (PMC4924825; doi:10.1371/journal.pone.0158052)
Supplement: S2 Table — (DOCX) [file pone.0158052.s004.docx]

**S2** **Table** QTL for days to heading in spray and spawn experiments

| **QTL** | **Position** | **Left marker** | **Right marker** | **Spray inoculation** | | |  | **Spawn inoculation** | | | **High DH** | **Association** |
| --- | --- | --- | --- | --- | --- | --- | --- | --- | --- | --- | --- | --- |
|  |  |  |  | **2011** | **2012** | **Mean** |  | **2011** | **2012** | **Mean** |  |  |
| 2BS | 64.1-72.5 | Bw_c8734_91 | Bw_c47608_273 | 15.5 | 5.9 | 5.9 |  |  |  |  | N |  |
| 2DS | 31.4-31.6 | BS00093760_51 | tplb0030j08_1960 | 21.7 | 22.9 | 22.1 |  |  |  |  | N |  |
| 4AL | 101.2-102.6 | Ex_c11968_204 | RAC875_c35979_263 |  |  |  |  | 15.8 | 24.9 | 22.0 | N | FHBs, FHBp |
| 5AL.1 | 167.2-170.8 | Vrn-A1 | w_Ku_c6977_12078885 | 14.1 | 16.0 | 15.9 |  | 20.3 | 30.9 | 28.0 | S | FHBs, FHBp, FDK, DON |
| 5AL.2 | 237.1-238.9 | Kukri_c49033_52 | wExc23795_33033959 |  |  |  |  | 11.4 | 7.1 | 8.2 | N |  |
| Accumulated percentage of variation explained | | | | 51.3 | 44.8 | 43.9 |  | 47.5 | 62.9 | 58.2 |  |  |

The percentage of explained phenotypic variation in the multiple regression models is shown

QTLs with LOD values higher than 3 are listed

*N* Naxos, *S* Soru#1, *FHBs* FHB index or severity after spray or spawn inoculation, *FHBp* FHB severity after point inoculation
