# Supplementary material for: QTL Characterization of Fusarium Head Blight Resistance in CIMMYT Bread Wheat Line Soru#1
Source: PLoS One. 2016 Jun 28;11(6):e0158052. doi: 10.1371/journal.pone.0158052 (PMC4924825; doi:10.1371/journal.pone.0158052)
Supplement: S3 Table — (DOCX) [file pone.0158052.s005.docx]

**S3** **Table** Sequence information of the two KASP markers for SNPs flanking the 2DLc QTL

| **SNP** | **Primer ID** | **FAM primer** | **VIC primer** | **Common primer** |
| --- | --- | --- | --- | --- |
| Kukri_c36639_186 | IWB44589 | TCTGCTAGAGGAGACATCACG | TCTGCTAGAGGAGACATCACA | TGTGCTCTGACTGACTTGATT |
| Excalibur_c7282_512 | IWB28643 | CGGTGACAAGGCTCTCTTCG | CGGTGACAAGGCTCTCTTCA | TGTTGCTGGAGATGTTCTCGT |

Cycling conditions: 94 ˚C for 15 min, followed by 10 cycles of a touchdown program of 94 ˚C for 20 s, 65-57 ˚C for 1 min, 72 ˚C for 30 s; then come 26 cycles of 94 ˚C for 20 s, 57 ˚C for 1 min, 72 ˚C for 30 s; with a final extension at 72 ˚C for 2 min.
